# Lactylproteome analysis indicates histone H4K12 lactylation as a novel biomarker in triple-negative breast cancer

**DOI:** 10.3389/fendo.2024.1328679

**Published:** 2024-05-08

**Authors:** Zhaolei Cui, Yanhong Li, Yingying Lin, Chaoqiang Zheng, Lingqing Luo, Dan Hu, Yan Chen, Zhenzhou Xiao, Yang Sun

**Affiliations:** ^1^ Laboratory of Biochemistry and Molecular Biology Research, Department of Laboratory Medicine, Clinical Oncology School of Fujian Medical University, Fujian Cancer Hospital, Fuzhou, China; ^2^ Department of Gynecology, Clinical Oncology School of Fujian Medical University, Fujian Cancer Hospital, Fuzhou, China; ^3^ Department of Pathology, Clinical Oncology School of Fujian Medical University, Fujian Cancer Hospital, Fuzhou, China

**Keywords:** triple negative breast cancer, histone H4, lactylation, biomarker, prognosis

## Abstract

**Objective:**

The established link between posttranslational modifications of histone and non-histone lysine (K) residues in cell metabolism, and their role in cancer progression, is well-documented. However, the lactylation expression signature in triple-negative breast cancer (TNBC) remains underexplored.

**Methods:**

We conducted a comprehensive lactylproteome profiling of eight pairs of TNBC samples and their matched adjacent tissues. This was achieved through 4-Dimensional label-free quantitative proteomics combined with lactylation analysis (4D-LFQP-LA). The expression of identified lactylated proteins in TNBC was detected using immunoblotting and immunohistochemistry (IHC) with specific primary antibodies, and their clinicopathological and prognostic significance was evaluated.

**Results:**

Our analysis identified 58 lactylation sites on 48 proteins, delineating the protein lactylation alteration signature in TNBC. Bioinformatic and functional analyses indicated that these lactylated proteins play crucial roles in regulating key biological processes in TNBC. Notably, lactylation of lysine at position 12 (H4K12lac) in the histone H4 domain was found to be upregulated in TNBC. Further investigations showed a high prevalence of H4K12lac upregulation in TNBC, with positive rates of 93.19% (137/147) and 92.93% (92/99) in TNBC tissue chip and validation cohorts, respectively. H4K12lac expression correlated positively with Ki-67 and inversely with overall survival (OS) in TNBC (HR [hazard ratio] =2.813, 95%CI [credibility interval]: 1.242-6.371, *P*=0.0164), suggesting its potential as an independent prognostic marker (HR=3.477, 95%CI: 1.324-9.130, *P*=0.011).

**Conclusions:**

Lactylation is a significant post-translational modification in TNBC proteins. H4K12lac emerges as a promising biomarker for TNBC, offering insights into the lactylation profiles of TNBC proteins and linking histone modifications to clinical implications in TNBC.

## Introduction

According to data from the International Agency for Research on Cancer (IARC) of the World Health Organization (WHO), breast cancer (BC) has now become the most common malignant tumor globally, surpassing lung cancer (1, 2). Triple-negative breast cancer (TNBC), characterized by the absence of estrogen receptor (ER), progesterone receptor (PR), and human epidermal growth factor receptor 2 (HER2) expression, represents approximately 15%–20% of all BC cases (3, 4). TNBC poses a greater clinical challenge compared to other BC types due to its higher rates of recurrence and metastasis, shorter median survival, and poorer prognosis (3, 5, 6). A reliable biomarker for clinical diagnosis, treatment, and prognostic evaluation of TNBC remains unidentified.

Malignant tumors, including BC, adaptively alter various cellular metabolic pathways during their development and progression to support growth, proliferation, and enhanced bioenergy and metabolic synthesis (7–9). In this context, signal transduction can occur during tumor cell metabolism through the chemical modification of proteins, including histones and non-histone proteins (10, 11). The post-translational modification of lysine (K) in histones or non-histones is believed to significantly influence cellular metabolism and regulate the tumor microenvironment (12, 13). First identified in 2019 by Zhao’s group, lactylation is a novel post-translational modification of lysine, actively regulated by the glycolytic metabolite lactic acid (14). Mass spectrometry revealed a mass shift in lysine residues in human BC cells, consistent with the addition of a lactyl group, leading researchers to propose lactate as a key mediator (14, 15). Dong et al.’s recent study on Escherichia coli demonstrated a distinct protein profile of lysine lactylation and showed that YdiF can catalyze the formation of lactyl-coenzyme A, a donor for lysine lactylation (16). Lactylation is intimately linked with tumors and may be involved in metabolic alterations, epigenetic modifications, immune evasion, and immunosurveillance (17–19).

Histones, comprising core histones (H2A, H2B, H3, and H4) and linker histones (H1 and H5), are alkaline, positively charged proteins and a fundamental component of chromatin (20, 21). The amino terminus of histones undergoes covalent modifications, altering chromatin structure (22, 23), leading to either transcriptional activation or gene silencing (24). Beyond gene expression regulation, histone posttranslational modifications also recruit protein complexes, impacting cell division, apoptosis, memory formation, and the immune and inflammatory responses (25, 26). Histone lactylation plays a role in tumorigenesis and progression (17, 27), such as facilitating the expression of the m^6^A reader protein YTHDF2 (YTH N6-methyladenosine RNA binding protein F2) (27). However, data on protein lactylation signatures in TNBC are currently limited. Employing 4-Dimensional label-free quantitative proteomics with lactylation analysis (4D-LFQP-LA) via liquid chromatography-mass spectrometry, we characterized differential protein lactylation expression profiles in TNBC. This analysis confirmed the upregulation of histone H4 lactylation at lysine position 12 (histone H4K12lac). Further validation of histone H4K12lac expression in TNBC and its clinical applications was conducted using an anti-L-lactyl-histone H4 (Lys 12) primary antibody. This study serves as a reference for ongoing research on histone lactylation in TNBC, assessing its clinical significance.

## Materials and methods

### Study design

Using 4D-LFQP-LA, we analyzed differentially expressed lactylation sites in TNBC samples and paired tumor-adjacent tissues (eight cases). Protein expression was quantified in thirteen cases using 4-Dimensional label-free quantitative proteomics (4D-LFQP). The accuracy of lactylproteome expression was verified through IHC and immunoblotting with specific antibodies targeting the identified lactylated site (histone H4K12lac). We further validated the expression of histone H4K12lac in TNBC and its association with clinicopathological features using a high-throughput tissue chip and IHC. Additionally, we evaluated the expression and prognostic significance of histone H4K12lac in TNBC using 99 TNBC samples and 71 paired tumor-adjacent tissues.

### Samples and clinical data

This study involved 112 patients with TNBC, including 84 paired adjacent non-cancer controls, treated at the Fujian Cancer Hospital (Fujian Branch of Fudan University Shanghai Cancer Center) between March 1^th^, 2018, and June 30^th^, 2023. All participants were women, averaging 56 years of age. Diagnoses were confirmed histopathologically, encompassing various histological types such as invasive ductal carcinoma, and non-specific invasive carcinomas, all negative for ER, PR, and HER2 expression. The follow-up period extended from March 10^th^, 2018, to August 31^th^, 2023. The Fujian Cancer Hospital Ethics Committee approved this study (ethical approval certificate: No. SQ2018-015-01). The high-throughput tissue chip included 150 TNBC cases and 30 tumor-adjacent tissues (Cat.no.HBreD180Bc01-1, SHANGHAI OUTDO BIOTECH, Shanghai, China), all diagnosed histopathologically. The samples were collected from female patients, with an average age of 52 years, and included histological types like invasive ductal carcinoma (IDC) (n=42), non-specific invasive carcinoma (NIDC) (n=96), medullary carcinoma (n=4), metaplastic carcinoma (n=3), mixed metaplastic carcinoma (n=1), basal-like carcinoma (n=1), squamous cell carcinoma (n=1), adenoid cystic carcinoma (n=1), and mucinous carcinoma (n=1). All tissue samples were collected without any intervention, and informed consent was obtained before sample collection.

### Main reagents

EpiQuik Total Histone Extraction Kit (Catalog.OP-0006, Epigentek, USA), anti-L-Lactyl-Histone H4 (Lys 12) rabbit monoclonal antibody [Cat no. PTM-1411RM, PTM BIO, Hangzhou, China; with technical support from Zhao’s group (14)]; anti-H4 rabbit monoclonal antibody (Product no. AF2581), and Enhanced BCA Protein Assay Kit (Product no. P0010S) were sourced from Beyotime Biotechnology (Shanghai, China). EliVisionTM Plus Two-Step Detection Kit (Product no. KIT-9903, Maixin, Fuzhou China).

### 4D-label free lactylproteome expression analysis

The samples underwent four rounds of treatment with a powder lysis buffer (1% Triton X-100, 1% protease inhibitor) and were subjected to ultrasonic lysis. After centrifugation at 4°C and 12000 × *g* for 10 min, the supernatant was transferred to a new centrifuge tube. Protein content was determined using a BCA Kit. Equal amounts of enzyme hydrolyzed each protein sample, and lysate volumes were standardized. The samples were mixed with an equal volume of precooled acetone, vortexed, followed by the addition of four volumes of precooled acetone, and precipitated at -20°C for 2 h. The precipitate was then centrifuged at 4500 × *g* for 5 min, washed two to three times with precooled acetone, and dried. Tetraethyl-ammonium bromide (TEAB) was added to achieve a final concentration of 200 mM, and the precipitate was dispersed using ultrasound. Trypsin was then added at a 1:50 protease:protein ratio (m/m) for overnight enzymolysis. Subsequently, the samples were treated with dithiothreitol (DTT) at 56°C for 30 min to achieve a final concentration of 5 mM, followed by the addition of IAA to reach a final concentration of 11 mM. Samples were incubated for 15 min at room temperature in the dark. Peptide fragments were dissolved in an IP buffer solution (100 mM NaCl, 1 mM EDTA, 50 mM Tris-HCl, 0.5% NP-40, pH 8.0), and the supernatant was transferred to a prewashed resin (antibody resin, PTM1404, PTM Bio), placed on a rotary shaker at 4°C, and incubated overnight. The resin-bound peptide was eluted thrice with 0.1% trifluoroacetic acid.

The peptides were dissolved in liquid chromatography mobile phase A and separated using a NanoElute ultraperformance liquid system. Mobile Phase A consisted of an aqueous solution with 0.1% formic acid and 2% acetonitrile, while Mobile Phase B was an acetonitrile-aqueous solution with 0.1% formic acid. Liquid gradient settings were as follows: 0-42 min, 6% -22% B; 42-54 min, 22% -30% B; 54-57 min, 30%-80% B; 57-60 min, 80% B; at a flow rate of 450 nL/min. The peptides, separated using an ultra-high-performance liquid phase system, were ionized in a capillary ion source and analyzed using timsTOF Pro 2 mass spectrometry (Bruker), with a secondary mass spectrum scan range of 100–1700. Data acquisition employed the Parallel Accumulation Serial Fragmentation (PASEF) model. PTM BIO (Hangzhou, China) provided technical support for this analysis.

### Bioinformatics analysis of lactylated proteins

The mass spectrometry-derived raw data sets underwent a comprehensive analytical process outlined as follows: 1) Initial quality control assessment of peptide and protein concentrations was performed based on database search outcomes; 2) Quantitative analysis of post-translational modification sites, including distribution and reproducibility, was conducted. Data showing quantitative intensity values across different samples were visually represented; 3) Functional annotation of identified proteins utilized various databases and classifications, such as Gene Ontology (GO), Kyoto Encyclopedia of Genes and Genomes (KEGG), protein domains, Clusters of Orthologous Groups/Eukaryotic Orthologous Groups (COG/KOG), and subcellular localization. Additionally, motif analysis specific to lactylation sites was carried out; 4) Inferential statistics, including fold change (FC) and T-test P-values, were calculated based on quantitative data. Differences were identified based on set threshold criteria, and one-way Analysis of Variance (ANOVA) was used to determine statistical significance across multiple groups; 5) Proteins differing between comparative groups were then classified by functional metrics, including GO secondary classification, subcellular localization stratification, COG/KOG categorization, and KEGG pathway analysis; and 6) Fisher’s exact test was applied to identify differences between the two groups. Functional categories analyzed included GO, KEGG, protein domains, Reactome, and Wikipathways.

To ensure high-quality analytical conclusions, the data set required further refinement. A False Discovery Rate (FDR) of 1% was set for identification parameters at spectrum, peptide, and protein levels. The primary bioinformatics tools used were based on R software, with a statistical significance threshold set at P<0.05. These analyses are supported by resources from PTM BIO (Hangzhou, China).

### 4D-LFQP analysis

The extraction and processing steps for 4D-LFQP analysis mirrored those of the 4D-LFQP-LA analysis. During liquid chromatography-mass spectrometry, the liquid gradient settings were 0–70 min, 6–24% B, 70–82 min, 24–32% B, 82–86 min, 32–80% B, 86–90 min, 80% B, with approximate ranges. The flow rate was consistently maintained at 450 nL/min. Technical support for this analysis was provided by PTM BIO (Hangzhou, China).

### IHC

All tissue samples were embedded in paraffin. The TNBC high-throughput tissue microarray (Cat.no.HBreD180Bc01-1) included 150 TNBC tissue spots and 30 tumor-adjacent tissue points. The EliVisionTM Plus two-step detection kit (Product no. KIT-9903 Fuzhou Maixin) was used for IHC analysis according to the kit’s instructions. Human anti-L-Lactyl-Histone H4 (Lys 12) rabbit mAb (Cat No. PTM-1411RM) was diluted to 1:100. H4 protein (1:1000) served as the positive control, and PBS as the negative control. Two pathologists conducted a double-blind evaluation of the staining results based on the intensity of staining and the proportion of positive staining in sections (28, 29). Patients were categorized into high expression groups (ranging from + to +++ or expression scores of 3–9) and low/negative expression groups (negative to ± or expression scores of 0–2), based on the intensity score of histone H4K12lac protein expression.

### Extraction of histones

The EpiQuik™ Total Histone Extraction Kit was used as per the manufacturer’s instructions. Briefly, tissue samples were processed using a Dounce homogenizer after being weighed and cut into small pieces (1–2 mm^3^) using a scalpel or scissors. A1:10 dilution of 10× Pre-Lysis Buffer with distilled water was prepared (e.g., 1 mL of 10× Pre-Lysis Buffer + 9 mL of water). Tissue fragments were dissociated using 50–60 strokes after adding 1 mL of diluted 1× Pre-Lysis Buffer per 200 mg of tissue. The homogenized mixture was then centrifuged at 4°C at 3000 rpm for 5 min in a 15 mL conical tube. For mixtures less than 2 mL, centrifugation was performed at 10000 rpm for 1 min at 4°C, and the supernatant was discarded.

### Immunoblotting

Following cellular lysis, histone proteins were isolated using the EpiQuik Total Histone Extraction Kit. Protein purity and concentration were determined using the BCA Protein Assay Kit. SDS-PAGE with a 10% gel matrix was employed for protein separation, running for 120 min at 90V. The proteins were then transferred to a PVDF membrane, which was blocked with 5% non-fat milk at room temperature for two hours. Antibody concentrations used were anti-L-Lactyl-Histone H4LYS12 at 1:1500 and reference protein lamin B at 1:1000. The membrane was incubated at room temperature for two hours, followed by an overnight incubation at 4°C, before adding the secondary antibody and incubating for an additional three hours. Imaging was conducted using the BD Protein Imaging System, with band intensities quantitatively assessed using Image J software (version 1.8.0).

### Statistical analysis

Quantitative data were presented as mean ± standard deviation (SD) and analyzed using SPSS 16.0 software. One-way Analysis of Variance (ANOVA) was employed, supplemented by t-tests for pairwise comparisons and assessments of variance homogeneity. In cases of heteroscedasticity, variable transformation or rank sum tests were applied. The χ^2^ test was used to evaluate expression differences of histone H4K12lac between TNBC and adjacent non-malignant tissues. Survival analysis was performed with the Kaplan-Meier method and Log-rank tests. The Cox proportional hazards model assessed the impact of histone H4K12lac expression levels on overall survival (OS) in TNBC patients. A significance level (α) was set at 0.05, with P-values below this threshold deemed statistically significant. Graphpad software (version 8.0.2) facilitated graphical representation of the data.

## Results

### Identification and bioinformatics analysis of lactylated proteins in TNBC

**Figure 1:**
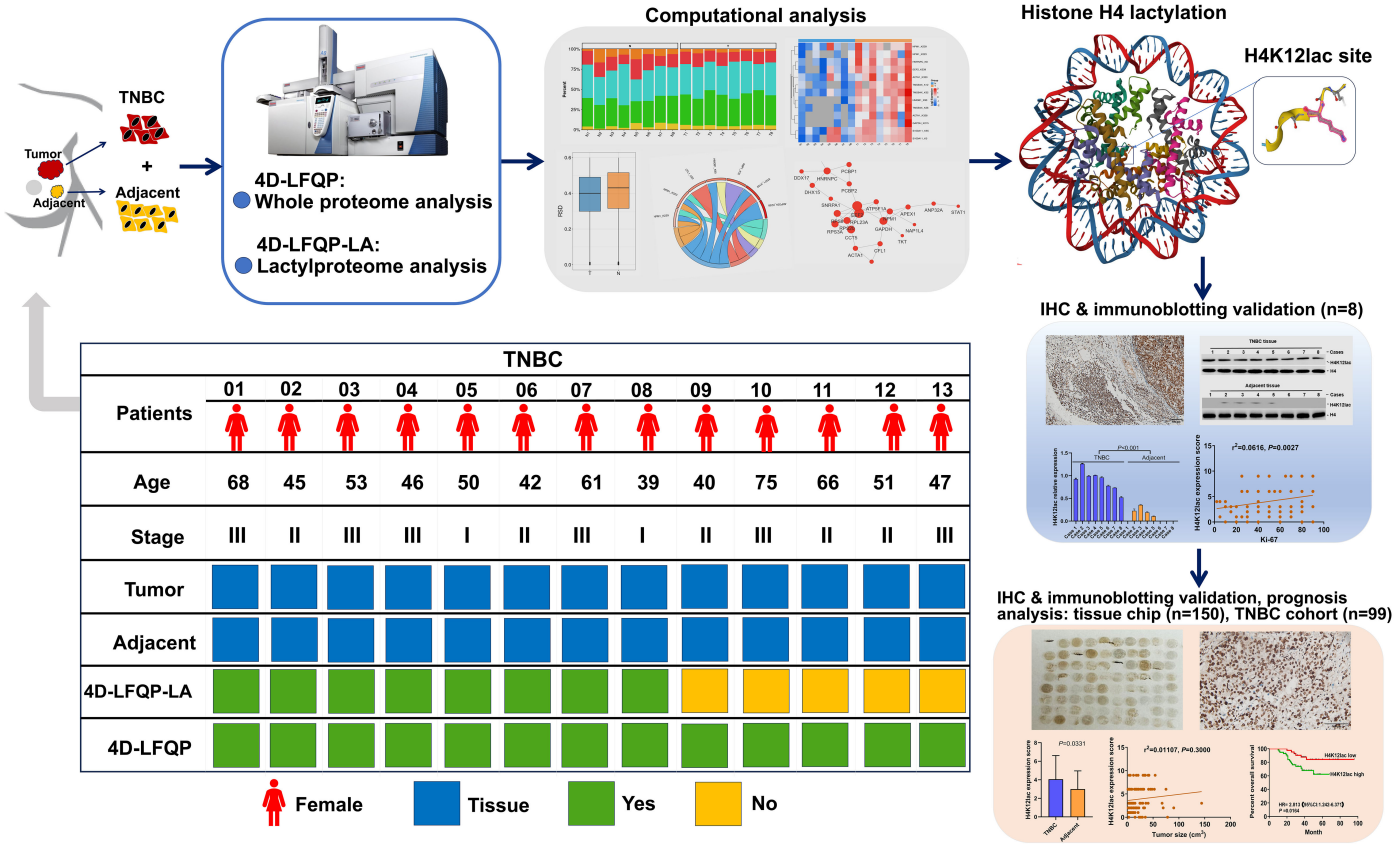
Schematic Overview of research design.

**Figure 2:**
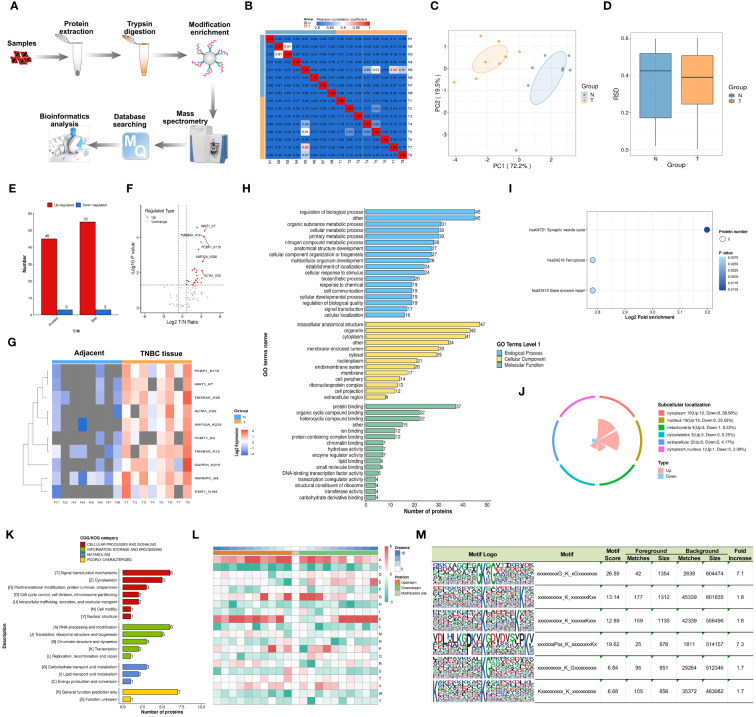
Characterization and Bioinformatics Profiling of Lactylation-Modified Proteins in TNBC. **(A)** Schematic representation of the 4D-FLQP-LA analytical workflow. **(B)** Heat map generated using Pearson correlation coefficient for pairwise sample comparison. **(C)** Bidimensional scatterplot depicting principal component analysis for protein quantification in replicate samples. **(D)** Box plot illustrating the Relative Standard Deviation (RSD) in protein quantification across repeated samples. **(E)** Categorization of identified lactylated proteins in TNBC based on effect size and statistical significance. **(F, G)** Volcano plot and heat map of key lactylated protein sites identified in TNBC. **(H)** Gene Ontology (GO) secondary classification. **(I)** Kyoto Encyclopedia of Genes and Genomes (KEGG) pathway analysis. **(J)** Proportional distribution of subcellular localization. **(K)** Clusters of Orthologous Groups/Eukaryotic Orthologous Groups (COG/KOG) functional categorization. **(L)** Motif analysis of lactylation-modified protein sites. **(M)** Conserved motif sites of lysine residues across six subtypes.

**Figure 1** illustrates the study’s flowchart. The process for detecting lactylproteome expression is shown in **Figure 2A**. Quality control analyses confirmed excellent quality of TNBC samples, with consistent quantitative results in biological duplicate samples (**Figures 2B-D**). Fifty-eight lactylation sites (55 up-regulated, three downregulated) were identified in 48 proteins (**Figure 2E**). **Figure 2F** displays all protein sites where lactylation occurs, and **Figure 2G** highlights 13 significant lactylation sites, including NPM1_K239, NPM1_K223, HNRNPC_K8, EEF2_K239, ACTA1_K330, TMSB4X_K12, TMSB4X_K32, HMGB1_K90, TMSB4X_K26, ACTA1_K328, GAPDH_K215, S100A11_K55, and S100A11_K3.

GO annotation analysis was used to identify cellular pathways, biological regulation mechanisms, and multicellular biological processes linked to these lactylated proteins. As depicted in **Figure 2H**, the primary biological processes involved regulation of biological processes, organic substance metabolic processes, and cellular metabolic processes. Key cellular components were the intracellular anatomical structure, organelles, and cytoplasm. Molecular functions included protein binding, organic cyclic compound binding, and heterocyclic compound binding. The KEGG database identified related pathways, with synaptic vesicle cycle, ferroptosis, and base excision repair being the primary signaling pathways (**Figure 2I**). Subcellular localization studies indicated that lactylated proteins were evenly distributed across the cytoplasm, nucleus, mitochondria, and cytoskeleton (39.58%, 39.58%, 8.33%, and 6.25%, respectively) (**Figure 2J**). The COG/KOG category showed higher abundance of lactylated proteins in signal transduction processes, RNA processing and modification, cytoskeleton, and translation, ribosomal structure and proteins (**Figure 2K**).

The motif features of the modification sites were analyzed using the MoMo tool based on the motif-x algorithm, with the heatmap presented in **Figure 2L**. Our investigation revealed that lactylation patterns in TNBC tissues were characterized by amino acid sequences around lactylated lysine, such as xxxxxxxxxG_K_xGxxxxxxxx, xxxxxxxxxx_K_xxxxxxxKxx, xxxxxxxxxx_K_xxxxxxKxxx, xxxxxxxPxx_K_xxxxxxxxKx, xxxxxxxxxx_K_Gxxxxxxxxx, and Kxxxxxxxxx_K_xxxxxxxxxx (**Figure 2M**).

### Functional analysis of lactylated proteins per Q cluster and quantitative proteomics analysis based on 4D-LFQP

**Figure 3:**
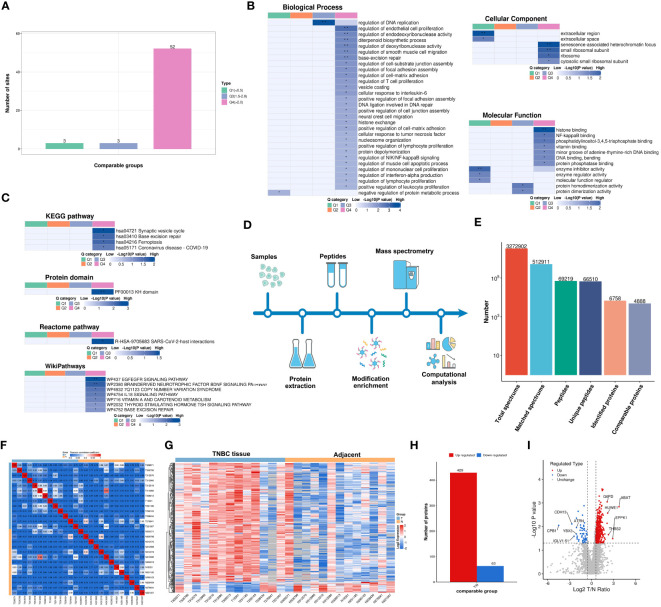
Functionality Stratification of Lactylated Proteins by Q Group Classification and 4-Dimensional Label-Free Quantitative Proteomics Analysis. **(A)** Lactylated proteins categorized into quartiles based on differential expression levels. **(B)** Heat map reflecting cluster analysis based on GO categorization, and classification of lactylated proteins according to **(C)** KEGG pathways, protein domain ontology, reactome pathways, and Wikipathways. **(D)** Analytical schema of the 4D-LFQP workflow. **(E)** Comprehensive enumeration of identified proteins, including peptide segments and protein count post-data filtration. **(F)** Heat map constructed using Pearson correlation coefficients between all thirteen paired samples. **(G)** Heat map showing differentially expressed protein sites identified in TNBC. **(H)** Histogram illustrating the identified differentially expressed protein sites. **(I)** Volcano plot detailing the spectrum of differentially expressed proteins in TNBC as determined by quantitative proteomics analysis.

Lactylation proteins were categorized into four groups based on differential expression: Q1 (downregulated < 0.5-fold), Q2 (downregulated 1.5-2-fold), Q3 (upregulated 1.5-2.0-fold), and Q4 (upregulated > 2.0-fold). Enrichment in KEGG pathways, protein domains, Reactome, and WikiPathways was analyzed for each group. Cluster analysis determined the correlation of protein functions with various differential expression levels in the comparison group. As shown in **Figure 3A**, three proteins were categorized as Q1, three as Q2, and 52 as Q4. The study primarily focused on the functional analysis of cluster Q4.

In cluster Q4, biological processes were predominantly associated with the regulation of DNA replication, endothelial cell proliferation, endodeoxyribonuclease activity, diterpenoid biosynthesis, deoxyribonuclease activity, smooth muscle cell migration, base excision repair, and T cell and lymphocyte proliferation (**Figure 3B**). Key cellular components included senescence-associated heterochromatin focus and small ribosomal subunit (**Figure 3B**). Molecular functions were chiefly related to histone binding (**Figure 3B**). KEGG pathway enrichment was significant in synaptic vesicle cycle, base excision repair, and ferroptosis (**Figure 3C**), along with enrichment in other protein domains, Reactome, and WikiPathways.

A 4D-LFQP analysis was performed on 26 samples, comprising 13 TNBC and paired cancer-adjacent samples. The workflow and sample quality control for 4D-LFQP are depicted in **Figures 3D-F**. A total of 492 differentially expressed proteins (DEPs) were identified, including 429 upregulated and 63 downregulated DEPs (**Figures 3G, H**). Ten significant proteins, namely ABAT, ATRN, CDH13, CPB1, EPPK1, G6PD, HUWE1, IGLV1-51, THBS2, and YBX3, were highlighted (**Figure 3I**). Notably, histone H1.5 was the only differentially expressed protein in the histone family in TNBC tissues (T/N ratio=1.6144, *P*=0.0276).

### Validation histone H4K12 lactylation in TNBC

**Figure 4:**
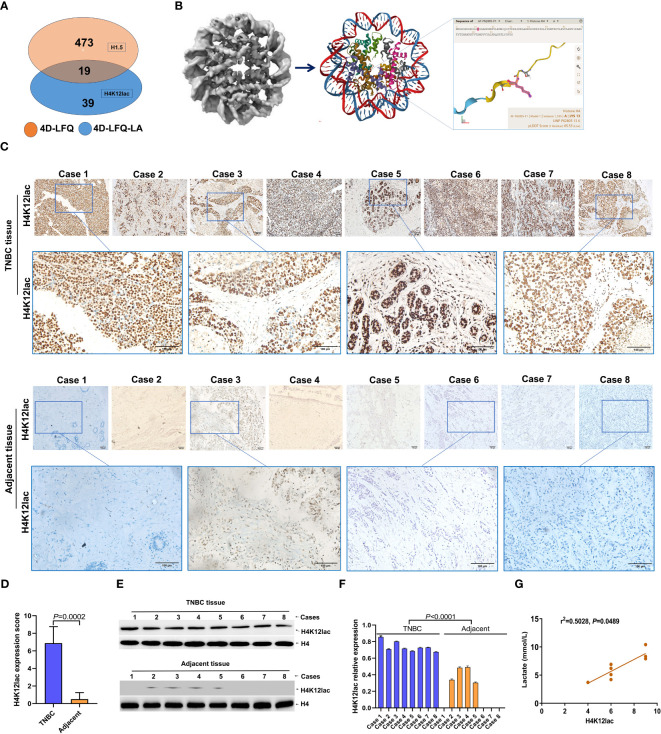
Empirical Validation of Histone H4K12lac Sites in Eight TNBC Cases. **(A)** Comparative identification of proteins through 4D-FLQP-LA and 4D-LFQP methods, noting H4K12lac expression alterations exclusively in lactylation levels. **(B)** Structural illustration of Histone H4 and the specific location of the lactylated H4K12lac site on the lysine residue. **(C, D)** Immunohistochemical (IHC) analysis demonstrating the expression and subcellular localization of lactylated H4K12lac in the eight TNBC cases. **(E, F)** Quantitative assessment and expression of lactylated H4K12lac in TNBC cases, as determined by immunoblotting and normalized to nuclear H4 using Image J software. **(G)** Positive correlation observed between lactylated H4K12lac and plasma lactate levels.

In the validation study, **Figure 4A** illustrates that 19 proteins were identified as common between the 4D-FLQP-LA and 4D-LFQP methods. The 4D-LFQP-LA analysis highlighted that histone H4 is characterized by differential lactylation of lysine at position 12 (histone H4K12lac; T/N ratio=4.489, *P*=0.0275) (**Figure 4B**). In contrast, the 4D-LFQP analysis indicated no significant difference in histone H4 protein expression between TNBC and adjacent tissues, suggesting that the elevation in histone H4K12lac is specific to its lactylation level. Validation of histone H4K12lac expression was conducted through IHC and immunoblotting on eight pairs of TNBC and para-cancer tissues from the 4D-LFQP-LA analysis. IHC results demonstrated predominant nuclear expression of histone H4K12lac in TNBC cells, with higher expression intensity in cancer tissues compared to para-cancer tissues (**Figures 4C, D**, *P*=0.0002). Immunoblotting further substantiated the elevated expression of histone H4K12lac in TNBC cases compared to adjacent controls (**Figures 4E, F**, *P*<0.0001). Subsequent studies showed a positive correlation between histone H4K12lac expression and plasma lactate levels (**Figure 4G**, *P*=0.0489), aligning with the 4D-LFQP-LA findings.

### Expression of lactylated histone H4K12 in TNBC tissue chip

**Figure 5:**
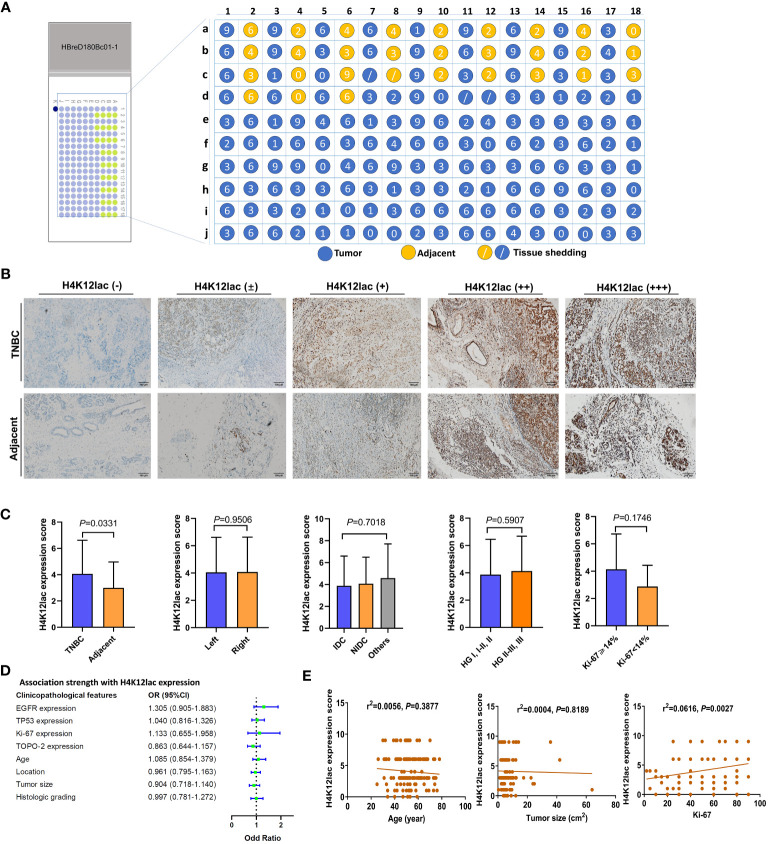
High-Throughput Validation of Lactylated Histone H4K12lac Expression in TNBC Tissue Array. **(A)** Quantitative scoring of lactylated H4K12lac expression in each sample on the TNBC tissue chip (n=180). **(B)** Analysis of lactylated H4K12lac expression in TNBC versus adjacent tissues using IHC. **(C)** Evaluation of lactylated H4K12lac expression relative to various clinicopathological parameters. **(D)** Assessment of the association strength between different clinicopathological factors and increased H4K12lac expression. **(E)** Correlative analyses. TNBC: triple-negative breast cancer; IDC: Invasive ductal carcinoma; NIDC: non-specific invasive carcinoma; HG: Histologic grading; Ki-67: antigen identified by monoclonal antibody Ki-67.

Further investigation of histone H4K12lac expression using a high-throughput TNBC tissue chip (Cat.no.HBreD180Bc01-1) revealed a positive expression rate of 93.19% (137/147) in cancer tissues, with 73.47% (108/147) showing high expression and 26.53% (39/147) displaying low/negative expression. In tumor-adjacent tissues, the positive expression rate was 89.66% (26/29), with high expression in 55.17% (16/29) and low/negative expression in 44.83% (13/29) of cases. This detailed expression profile is depicted in **Figures 5A, B**. The expression score in cancer tissues was significantly higher than in adjacent tissues (*P*=0.0331), but there were no significant differences based on location (left vs. right), cancer subtype (IDC vs. NIDC vs. others), or histologic grading (I, I-II, II vs. II-III, III) (**Figure 5C**). Correlation analyses indicated no association between histone H4K12lac expression and age, TP53, EGFR, TOPO-2 expression, tumor location, size, or histologic grading (**Table 1**; **Figures 5D, E**). Intriguingly, H4K12lac expression correlated with Ki-67 levels (**Figure 5E**), although the expression score in the Ki-67≥14% group was equivalent to that in the Ki-67 <14% group (**Figure 5C**).

**Table 1:** Clinicopathological Characteristics Pertaining to H4K12lac Expression in TNBC, Ascertained via Tissue Chip.

Clinicopathological features	Total cases	H4K12lac low (Negative ~ ±)	H4K12lac high (+ ~ +++)	χ^2^ value	*P* value
Age	138*(Data missing, n=10)			0.465	0.495
≥50	86* (Tissue shedding, n=1)	25	61		
<50	52* (Tissue shedding, n=1)	18	34		
Location	147*			0.162	0.688
Left	81* (Tissue shedding, n=1)	22	59		
Right	66* (Tissue shedding, n=2)	16	50		
Tumor size	141* (Data missing, n=6)			0.633	0.462
>2 cm	108* (Tissue shedding, n=2)	34	74		
≤2 cm	33* (Tissue shedding, n=1)	8	25		
Histologic grading	147*			0.001	0.98
I+II	39	12	27		
III+IV	108*(Tissue shedding, n=3)	33	75		
TOPO-2 expression	125*(Missing data n=23)			1.103	0.294
Negtive+I	36* (Tissue shedding, n=1)	14	22		
II+III+IV	89*(Tissue shedding, n=1)	26	63		
Ki-67 expression	145*(Missing data n=2)			0.01	0.919
≥14%	137*(Tissue shedding, n=3)	40	97		
<14%	8	3	5		
TP53 expression	130*(Missing data n=18)			0.105	0.746
Postive	86 (Tissue shedding, n=2)	25	61		
Negative	44	14	30		
EGFR expression	129*(Missing data n=19)			2.787	0.095
Postive	104 (Tissue shedding, n=2)	28	76		
Negative	25	11	14		
Cancer subtype	148			0.295	0.863
IDC	40* (Tissue shedding, n=2)	13	27		
NIDC	95* (Tissue shedding, n=1)	27	68		
Others	12	4	8		

IDC, Invasive ductal carcinoma; NIDC, non-specific invasive carcinoma. *Data missing or tissue sample shedding.

### Lactylated histone H4K12lac as a prognostic marker of TNBC

**Figure 6:**
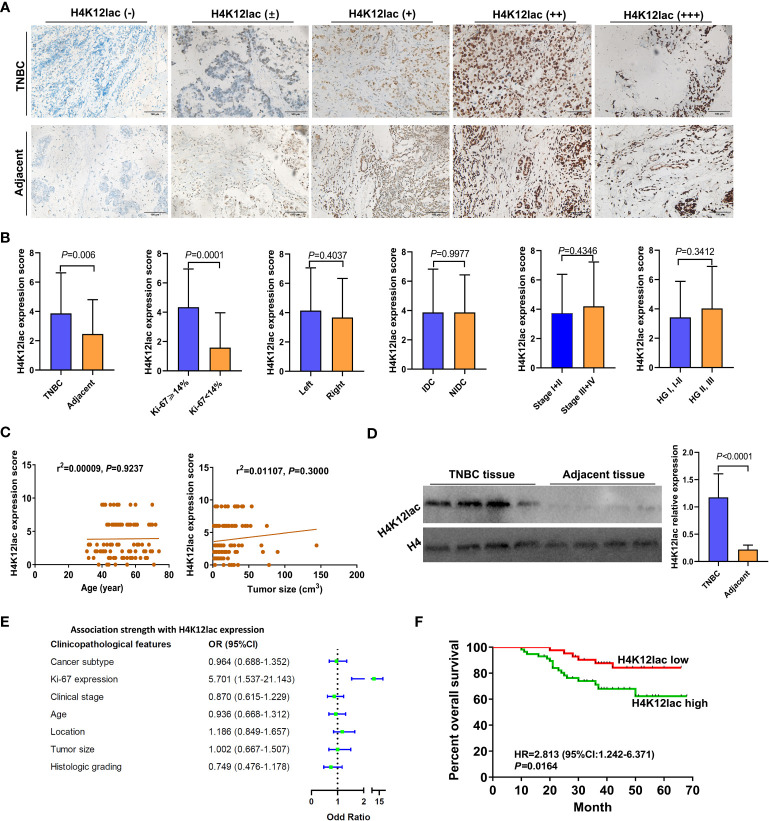
Prognostic Implications of Lactylated Histone H4K12lac in TNBC. **(A)** IHC-based analysis of lactylated H4K12lac expression in TNBC and adjacent tissues in the study cohort (n=99). **(B)** Profiling of H4K12lac expression across various clinicopathological parameters. **(C)** Linear regression-based correlation analyses. **(D)** Immunoblotting analysis of H4K12lac in four paired TNBC and adjacent tissue samples (n=4), normalized against H4 expression. **(E)** Forest plot illustrating the association strength between different clinicopathological parameters and elevated H4K12lac expression. **(F)** Negative correlation of elevated H4K12lac expression with overall survival (OS). TNBC: triple-negative breast cancer; IDC: Invasive ductal carcinoma; NIDC: non-specific invasive carcinoma; HG: Histologic grading; Ki-67: antigen identified by monoclonal antibody Ki 67.

For thorough validation, a cohort of 99 TNBC cases and 71 paired adjacent controls was examined. Immunohistochemistry (IHC) analysis, as shown in **Figure 6A**, revealed that 92.93% (92/99) of TNBC cases exhibited histone H4K12lac expression, with 57 cases showing elevated expression, 35 with diminished expression, and 7 with no expression. Tissue microarray analysis confirmed significantly higher levels of histone H4K12lac in cancerous tissues compared to adjacent non-cancerous tissues (**Figure 6B**, *P*=0.006). Notably, histone H4K12lac expression was markedly higher in samples with Ki-67 ≥ 14% than in those with Ki-67 < 14% (**Figure 6B**, *P*=0.0001), and a significant association between Ki-67 and H4K12lac expression was observed (**Figure 6E**, OR [odds ratio] =5.701, 95%CI: 1.537-21.143; **Table 2**, χ2 = 15.443, *P*<0.001). However, no significant correlation was found between histone H4K12lac expression and patient age, tumor size, or other clinicopathological parameters (**Figures 6B, C**; **Table 2**). Immunoblotting further supported the increased expression of histone H4K12lac in TNBC tissue in 4 cases, with detection of low level in adjacent non-cancerous tissues (**Figure 6D**).

**Table 2:** Clinicopathological Characteristics of H4K12lac Expression in TNBC, Determined Through Validation Cohort.

Clinicopathological features	Total cases	H4K12lac low (Negative ~ ±)	H4K12lac high (+ ~ +++)	χ^2^ value	*P* value
Age				0.146	0.702
≥50	52	23	29		
<50	47	19	28		
Location				0.977	0.323
Left	41	15	26		
Right	58	27	31		
Tumor size				0	0.991
>2 cm	80	31	49		
≤2 cm	18	7	11		
Histologic grading				1.883	0.17
I, I-II	26	14	12		
II, III	73	28	45		
Clinical stage				0.584	0.445
I+II	69	31	38		
III+IV	30	11	19		
Ki-67 expression				15.443	<0.001
≥14%	82	27	55		
<14%	17	15	2		
Cancer subtype				0.044	0.834
IDC	53	23	30		
NIDC	46	19	27		

IDC, Invasive ductal carcinoma; NIDC, non-specific invasive carcinoma.

The median follow-up duration for the 99 patients with TNBC was 41 months (range: 10 to 66 months). Median OS durations for high and low/negative expression groups of histone H4K12lac were 35 and 49 months, respectively. Patients with high H4K12lac expression exhibited significantly shorter 5-year OS compared to those with low/negative expression (HR=2.813, 95%CI:1.242-6.371, *P*=0.0164) (**Figure 6F**). Cox proportional hazards regression analysis indicated that histone H4K12lac expression independently affected OS in TNBC patients (**Table 3**, HR=3.477, 95%CI: 1.324-9.130, *P*=0.011), signifying that high H4K12lac expression posed 3.477 times the death risk compared to low/negative expression.

**Table 3:** Cox Regression-Based Prognostic Risk Factor Analysis in a Cohort of 99 TNBC Patients.

Clinicopathological factor	β value	S.e value	Wald value	HR	95% CI	*P* value
Age (≥50 vs. <50)	0.065	0.458	0.020	1.067	0.435-2.617	0.887
Location (Right vs. Left)	-0.341	0.509	0.449	0.711	0.262-1.928	0.503
Cancer subtype (IDC vs. NIDC)	0.231	0.478	0.234	1.260	0.494-3.216	0.629
Histologic grading (I, I-II vs. II, III)	-1.292	1.092	1.401	0.275	0.032-2.333	0.236
Clinical stage (I+II vs. III+IV)	2.078	0.459	20.494	7.992	3.250-19.653	0.000
Ki-67 expression (≥14% vs. <14%)	-0.965	0.636	2.303	0.381	0.110-1.325	0.129
H4K12lac (High vs. Low)	1.246	0.493	6.399	3.477	1.324-9.130	0.011

HR, hazard ratio.

## Discussion

Historically, lactate was regarded as a mere metabolic product (30, 31). However, recent evidence suggests lactate production and the resultant acidification of the tumor microenvironment play crucial roles in angiogenesis, invasion, metastasis, and immune evasion in certain cancer (19, 30, 32). Intriguingly, further studies have validated that lactylation occurs in both histone and non-histone proteins and that protein lysine lactylation is vital for lactate function (4, 14, 22). Although histone lactylation has been primarily associated with non-neoplastic diseases (33, 34), its presence in cancer has been infrequently reported.

For the first time, we mapped the protein lactylation alteration signature in eight pairs of TNBC samples, identifying 58 lactylation sites, predominantly demonstrating an upregulation of lysine lactylation. Our bioanalytical results elucidated the roles of lactylated proteins and related signaling pathways in regulating biological, organic substance metabolic, and cellular metabolic processes. These lactylated proteins were found to be involved in the synaptic vesicle cycle, ferroptosis, and base excision repair. Cluster analysis indicated that 89.66% (52/58) of the sites exhibited more than a 2.0-fold increase in lactylation, predominantly associated with DNA replication and deoxyribonuclease activity regulation. The primary pathways involved were synaptic vesicle cycle, base excision repair, and ferroptosis. Notably, among the differentially expressed lactylation sites, histone H4K12lac was the only histone family protein to exhibit lactylation (upregulated T/N=4.489), with modification sites exclusively in the histone H4 protein functional region. However, 4D-LFQP analysis showed no difference in basal histone H4 expression between TNBC and adjacent tissues, indicating upregulation solely at the lactylation level. Similarly, a recent study identified significant expression of lactylated histone H3K18 in patients with septic shock, suggesting its potential as a diagnostic and severity marker (34). Thus, histone H4K12lac may represent a pathological protein modification in TNBC, with clinical significance for detection.

To validate the accuracy of histone H4K12lac site identification via 4D-LFQP-LA analysis, an anti-L-Lactyl-Histone H4 (Lys 12) was employed alongside IHC and immunoblotting in eight pairs of TNBC samples. We observed that lactylation modification of histone H4K12 was higher in cancer tissues compared to adjacent controls. Aligning with sequencing analysis, the basal expression level of histone H4 protein remained unchanged in TNBC versus controls. Histone H4K12lac was expressed in more than 90% of TNBC samples based on a high-throughput TNBC tissue microarray. Intriguingly, H4K12lac expression correlated positively with plasma lactate levels. The Warburg effect indicates that tumor cells metabolize glucose into lactic acid via glycolysis differently than normal cells (35, 36). As a cancer metabolite, lactate plays a crucial role in tumor proliferation, metastasis, angiogenesis, immune evasion, and the response to radiotherapy and chemotherapy (17, 37). Elevated lactate levels in TNBC (38), and lactylation’s dependency on lactate (14, 15), suggest that increased lactate may lead to higher histone H4K12lac expression in TNBC. The correlation of histone H3K18la protein expression with serum lactate levels in septic shock patients further substantiates our findings and hypothesis (34).

In this study, we explored the expression and prognostic value of histone H4K12lac in TNBC using case and follow-up data. Histone H4K12lac exhibited high expression levels in over 70% of TNBC cells, compared to about 50% in adjacent tissues. Follow-up data revealed that median OS durations were 35 months for the high expression group and 49 months for the low/negative expression groups of histone H4K12lac. Patients with high expression of histone H4K12lac in TNBC had a poorer prognosis than those with low or negative expression, establishing histone H4K12lac as an independent risk factor for OS in TNBC patients. Previous research has shown higher histone lactylation levels in tumor cells, correlating with poor prognosis in ocular melanoma (27), and bioinformatics analysis indicated that a prognostic model based on non-histone lactylation proteins in gastric cancer could be constructed, yielding AUCs greater than 0.70 over 1, 2, and 3 years (39). Our findings align with these studies, suggesting that lactylated proteins hold potential for evaluating tumor prognosis.

Our data revealed a notable positive correlation between H4K12lac and Ki-67 expression within our validation cohort. However, this correlation did not reach statistical significance in the tissue chip cohort for TNBC. Upon further examination, we found that the skewed distribution of Ki-67 levels on the tissue chip—over 90% with Ki-67 levels ≥14% and only eight cases with levels <14%—contributed to this outcome. By increasing the number of TNBC cases with Ki-67 levels <14%, we identified a positive correlation between H4K12lac and Ki-67 in these additional cases. Nevertheless, our study had several limitations, including a small sample size of only 16 TNBC and adjacent samples analyzed using 4D-LFQP-LA, leading to the potential for biased results. Additionally, histone H4K12lac was only detected using IHC and immunoblotting, and its relationship with BC molecular subtyping was not explored. Future research should focus on understanding the biological role of histone H4K12lac in TNBC and developing an ELISA kit for its detection.

In conclusion, this is the first study to map the differential lactylproteome expression signature in TNBC through 4D-LFQP-LA analysis. We confirmed the expression and prognostic importance of histone H4K12lac in TNBC, highlighting its potential as a biomarker. This study bridges the gap between histone modifications and their clinical utility in TNBC, but further research with a larger sample size is necessary to substantiate our findings.

## Data availability statement

The original contributions presented in the study are included in the article/supplementary material. Further inquiries can be directed to the corresponding authors.

## Ethics statement

The studies involving humans were approved by The Ethics Committee of Fujian Cancer Hospital. The studies were conducted in accordance with the local legislation and institutional requirements. The participants provided their written informed consent to participate in this study.

## Author contributions

ZC: Conceptualization, Funding acquisition, Writing – original draft. YHL: Data curation, Formal analysis, Writing – original draft. YYL: Investigation, Methodology, Writing – original draft. CZ: Formal analysis, Software, Writing – original draft. LL: Investigation, Writing – review & editing. DH: Methodology, Writing – review & editing. YC: Supervision, Writing – review & editing. ZX: Supervision, Validation, Writing – review & editing. YS: Conceptualization, Funding acquisition, Supervision, Writing – review & editing.

## References

[1] Ferlay J Colombet M Soerjomataram I Parkin DM Piñeros M Znaor A. Cancer statistics for the year 2020: an overview. Int J Cancer. (2021). doi: 10.1002/ijc.33588 33818764

[2] Sung H Ferlay J Siegel RL Laversanne M Soerjomataram I Jemal A. Global cancer statistics 2020: globocan estimates of incidence and mortality worldwide for 36 cancers in 185 countries. CA: Cancer J Clin. (2021) 71:209–49. doi: 10.3322/caac.21660 33538338

[3] Derakhshan F Reis-Filho JS. Pathogenesis of triple-negative breast cancer. Annu Rev Pathol. (2022) 17:181–204. doi: 10.1146/annurev-pathol-042420-093238 35073169 PMC9231507

[4] Rizzo A Cusmai A Acquafredda S Giovannelli F Rinaldi L Misino A. Keynote-522, impassion031 and geparnuevo: changing the paradigm of neoadjuvant immune checkpoint inhibitors in early triple-negative breast cancer. Future Oncol (London England). (2022) 18:2301–9. doi: 10.2217/fon-2021-1647 35378995

[5] Honda Y Aruga T Yamashita T Miyamoto H Horiguchi K Kitagawa D. Prolonged survival after diagnosis of brain metastasis from breast cancer: contributing factors and treatment implications. Japan J Clin Oncol. (2015) 45:713–8. doi: 10.1093/jjco/hyv067 25981620

[6] Carey L Winer E Viale G Cameron D Gianni L. Triple-negative breast cancer: disease entity or title of convenience? Nat Rev Clin Oncol. (2010) 7:683–92. doi: 10.1038/nrclinonc.2010.154 20877296

[7] Wang Z Jiang Q Dong C. Metabolic reprogramming in triple-negative breast cancer. Cancer Biol Med. (2020) 17:44–59. doi: 10.20892/j.issn.2095-3941.2019.0210 32296576 PMC7142847

[8] Boroughs LK DeBerardinis RJ. Metabolic pathways promoting cancer cell survival and growth. Nat Cell Biol. (2015) 17:351–9. doi: 10.1038/ncb3124 PMC4939711 25774832

[9] Martínez-Reyes I Chandel NS. Cancer metabolism: looking forward. Nat Rev Cancer. (2021) 21:669–80. doi: 10.1038/s41568-021-00378-6 34272515

[10] Chang S Yim S Park H. The cancer driver genes idh1/2, jarid1c/ kdm5c, and utx/ kdm6a: crosstalk between histone demethylation and hypoxic reprogramming in cancer metabolism. Exp Mol Med. (2019) 51:1–17. doi: 10.1038/s12276-019-0230-6 PMC6586683 31221981

[11] Toh TB Lim JJ Chow EK. Epigenetics in cancer stem cells. Mol Cancer. (2017) 16:29. doi: 10.1186/s12943-017-0596-9 28148257 PMC5286794

[12] Gil J Ramírez-Torres A Encarnación-Guevara S. Lysine acetylation and cancer: A proteomics perspective. J Proteomics. (2017) 150:297–309. doi: 10.1016/j.jprot.2016.10.003 27746255

[13] Ding P Ma Z Liu D Pan M Li H Feng Y. Lysine acetylation/deacetylation modification of immune-related molecules in cancer immunotherapy. Front Immunol. (2022) 13:865975. doi: 10.3389/fimmu.2022.865975 35585975 PMC9108232

[14] Zhang D Tang Z Huang H Zhou G Cui C Weng Y. Metabolic regulation of gene expression by histone lactylation. Nature. (2019) 574:575–80. doi: 10.1038/s41586-019-1678-1 PMC6818755 31645732

[15] Moreno-Yruela C Zhang D Wei W Bæk M Liu W Gao J. Class I histone deacetylases (Hdac1-3) are histone lysine delactylases. Sci Adv. (2022) 8:eabi6696. doi: 10.1126/sciadv.abi6696 35044827 PMC8769552

[16] Dong H Zhang J Zhang H Han Y Lu C Chen C. Yiac and cobb regulate lysine lactylation in escherichia coli. Nat Commun. (2022) 13:6628. doi: 10.1038/s41467-022-34399-y 36333310 PMC9636275

[17] Lv X Lv Y Dai X. Lactate, histone lactylation and cancer hallmarks. Expert Rev Mol Med. (2023) 25:e7. doi: 10.1017/erm.2022.42 36621008

[18] Sun L Zhang H Gao P. Metabolic reprogramming and epigenetic modifications on the path to cancer. Protein Cell. (2022) 13:877–919. doi: 10.1007/s13238-021-00846-7 34050894 PMC9243210

[19] Chen L Huang L Gu Y Cang W Sun P Xiang Y. Lactate-lactylation hands between metabolic reprogramming and immunosuppression. Int J Mol Sci. (2022) 23(19):11943. doi: 10.3390/ijms231911943 PMC9569569 36233246

[20] Crane-Robinson C. Linker histones: history and current perspectives. Biochim Biophys Acta. (2016) 1859:431–5. doi: 10.1016/j.bbagrm.2015.10.008 26459501

[21] Biterge B Schneider R. Histone variants: key players of chromatin. Cell Tissue Res. (2014) 356:457–66. doi: 10.1007/s00441-014-1862-4 24781148

[22] Zhang Y Sun Z Jia J Du T Zhang N Tang Y. Overview of histone modification. Adv Exp Med Biol. (2021) 1283:1–16. doi: 10.1007/978-981-15-8104-5_1 33155134

[23] Tolsma TO Hansen JC. Post-translational modifications and chromatin dynamics. Essays Biochem. (2019) 63:89–96. doi: 10.1042/ebc20180067 31015385

[24] Morgan MAJ Shilatifard A. Reevaluating the roles of histone-modifying enzymes and their associated chromatin modifications in transcriptional regulation. Nat Genet. (2020) 52:1271–81. doi: 10.1038/s41588-020-00736-4 33257899

[25] Ratajczak W Rył A Mizerski A Walczakiewicz K Sipak O Laszczyńska M. Immunomodulatory potential of gut microbiome-derived short-chain fatty acids (Scfas). Acta Biochim Polonica. (2019) 66:1–12. doi: 10.18388/abp.2018_2648 30831575

[26] Hedin CRH Sonkoly E Eberhardson M Ståhle M. Inflammatory bowel disease and psoriasis: modernizing the multidisciplinary approach. J Internal Med. (2021) 290:257–78. doi: 10.1111/joim.13282 33942408

[27] Yu J Chai P Xie M Ge S Ruan J Fan X. Histone lactylation drives oncogenesis by facilitating M(6)a reader protein ythdf2 expression in ocular melanoma. Genome Biol. (2021) 22:85. doi: 10.1186/s13059-021-02308-z 33726814 PMC7962360

[28] Hu D Cui Z Peng W Wang X Chen Y Wu X. Apelin is associated with clinicopathological parameters and prognosis in breast cancer patients. Arch gynecol obstet. (2022) 306:1185–95. doi: 10.1007/s00404-022-06433-3 35249152

[29] Cui Z Chen Y Hu M Lin Y Zhang S Kong L. Diagnostic and prognostic value of the cancer-testis antigen lactate dehydrogenase C4 in breast cancer. Clinica chimica acta; Int J Clin Chem. (2020) 503:203–9. doi: 10.1016/j.cca.2019.11.032 31794764

[30] Rabinowitz JD Enerbäck S. Lactate: the ugly duckling of energy metabolism. Nat Metab. (2020) 2:566–71. doi: 10.1038/s42255-020-0243-4 PMC7983055 32694798

[31] Hall MM Rajasekaran S Thomsen TW Peterson AR. Lactate: friend or foe. PM R J injury funct Rehabil. (2016) 8:S8–s15. doi: 10.1016/j.pmrj.2015.10.018 26972271

[32] Hirschhaeuser F Sattler UG Mueller-Klieser W. Lactate: A metabolic key player in cancer. Cancer Res. (2011) 71:6921–5. doi: 10.1158/0008-5472.can-11-1457 22084445

[33] Yao Y Bade R Li G Zhang A Zhao H Fan L. Global-scale profiling of differential expressed lysine-lactylated proteins in the cerebral endothelium of cerebral ischemia-reperfusion injury rats. Cell Mol Neurobiol. (2023) 43:1989–2004. doi: 10.1007/s10571-022-01277-6 36030297 PMC11412193

[34] Chu X Di C Chang P Li L Feng Z Xiao S. Lactylated histone H3k18 as a potential biomarker for the diagnosis and predicting the severity of septic shock. Front Immunol. (2021) 12:786666. doi: 10.3389/fimmu.2021.786666 35069560 PMC8773995

[35] Schwartz L Supuran CT Alfarouk KO. The Warburg effect and the hallmarks of cancer. Anti-cancer Agents med Chem. (2017) 17:164–70. doi: 10.2174/1871520616666161031143301 27804847

[36] López-Lázaro M. The Warburg effect: why and how do cancer cells activate glycolysis in the presence of oxygen? Anti-cancer Agents med Chem. (2008) 8:305–12. doi: 10.2174/187152008783961932 18393789

[37] Yang X Lu Y Hang J Zhang J Zhang T Huo Y. Lactate-modulated immunosuppression of myeloid-derived suppressor cells contributes to the radioresistance of pancreatic cancer. Cancer Immunol Res. (2020) 8:1440–51. doi: 10.1158/2326-6066.cir-20-0111 32917658

[38] Naik A Decock J. Lactate metabolism and immune modulation in breast cancer: A focused review on triple negative breast tumors. Front Oncol. (2020) 10:598626. doi: 10.3389/fonc.2020.598626 33324565 PMC7725706

[39] Yang H Zou X Yang S Zhang A Li N Ma Z. Identification of lactylation related model to predict prognostic, tumor infiltrating immunocytes and response of immunotherapy in gastric cancer. Front Immunol. (2023) 14:1149989. doi: 10.3389/fimmu.2023.1149989 36936929 PMC10020516

